# Influence of placental exosomes from early onset preeclampsia women umbilical cord plasma on human umbilical vein endothelial cells

**DOI:** 10.3389/fcvm.2022.1061340

**Published:** 2022-12-23

**Authors:** Mengqi Gu, Fengyuan Zhang, Xiaotong Jiang, Pengzheng Chen, Shuting Wan, Qingfeng Lv, Yuan Lu, Qian Zhou, Yanyun Wang, Lei Li

**Affiliations:** ^1^Department of Obstetrics and Gynaecology, Shandong Provincial Hospital, Shandong University, Jinan, Shandong, China; ^2^Department of Obstetrics and Gynaecology, Shandong Provincial Hospital Affiliated to Shandong First Medical University, Jinan, Shandong, China; ^3^Key Laboratory of Birth Regulation and Control Technology of National Health and Family Planning Commission of China, Maternal and Child Health Hospital of Shandong Province, Jinan, China; ^4^The Laboratory of Medical Science and Technology Innovation Center (Institute of Translational Medicine), Shandong First Medical University (Shandong Academy of Medical Sciences) of China, Jinan, China

**Keywords:** placental exosomes, HUVECs, hypoxia/reoxygenation, permeability of the endothelial monolayer, early onset preeclampsia, pregnancy

## Abstract

**Background:**

Early onset preeclampsia (EOSP, PE) is characterized by hypertension, proteinuria, and endothelial dysfunction. Oxidative stress-induced trophoblast dysfunction is a major pathology in PE. Placental exosomes are extracellular vesicles that are involved in “mother-placenta-foetal communication” and can regulate the biological functions of endothelial cells. Our study was designed to evaluate placental exosomes effects on endothelial cells.

**Methods:**

Umbilical cord blood from normal pregnant women and patients with PE were collected. A hypoxia/reoxygenation (H/R) model in human first trimester extravillous trophoblast cell (HTR8/SVneo) line to simulate the PE model of oxidative stress *in vitro*. Then, placental exosomes (i.e., NO-exo, H/R-exo, N-exo, and PE-exo) were extracted and identified. Finally, the effects of placental exosomes on the biological functions of human umbilical vein endothelial cells (HUVECs) were further evaluated by performing a series of experiments.

**Results:**

Placental exosomes had a double-membrane cup structure with diameters of 30–150 nm, and there was no obvious difference in placental exosomes. Compared with NO-exo and N-exo, H/R-exo and PE-exo inhibited HUVECs proliferation, tube formation and migration, increased permeability and apoptosis *in vitro*.

**Conclusion:**

We hypothesize that H/R-exo and PE-exo impair vessel development by disrupted biological functions in endothelial cells, which may result in vascular disorders in offspring.

## 1 Introduction

Early onset preeclampsia (PE) is a clinical syndrome characterized by hypertension, proteinuria, and endothelial dysfunction (1). PE can cause adverse outcomes, such as miscarriage, preterm birth and fetal growth restriction, and may lead to increased maternal and fetal morbidity (2, 3). The pathogenesis of PE is not clearly understood, thus, clarifying its pathogenesis is essential for subsequent treatment.

Endothelial dysfunction is the leading cause of PE, and can cause hypertension, proteinuria, and other conditions (1). Human umbilical vein endothelial cells (HUVECs) isolated from the umbilical cords of PE patients showed changes in the expression and distribution of endothelial connexins, resulting in increased endothelial monolayer permeability, compared with umbilical cord cells isolated from normal pregnant women (4). The offspring of women with PE are at increased risk of cardiovascular disease (5). Throughout pregnancy, oxidative stress in the placenta may lead to pregnancy complications. Placental oxidative stress is a crucial step in the pathogenesis of PE, and the mechanism of placental oxidative stress in PE may involve hypoxia and reoxygenation (H/R) (6). Adequate oxygen supply is essential for normal metabolism, growth and development in the human body during the embryonic, fetal and postnatal stages. In early pregnancy, inadequate trophoblast invasion of the maternal spiral artery leads to reduced placental perfusion and the release of many biological factors, which can lead to endothelial injury and acute maternal syndrome with systemic multiple organ failure, causing preeclampsia (7). H/R cause changes in cellular structure and function, which can result in miscarriage, intrauterine growth restriction (IUGR) and premature membrane rupture, increasing the risk of premature delivery and postpartum infection (8). H/R can cause a variety of pathological reactions, which can activate endoplasmic reticulum stress, cell dysfunction and cell death (9).

Mother-placenta-fetal communication is important for maintaining a normal pregnancy. Exosomes are extracellular vesicles ranging from 30–150 nm in diameter (10). Placental exosomes are an important intercellular communication factor, for example, in PE, placental exosomes inhibit eNOS expression in HUVECs (11). Placental exosomes may play critical roles in the functions of the placenta and endothelial cells, the fetus and the mother (12–14). Higher levels of plasma exosomes have been measured in pregnant women than in non-pregnant women (15). It has been shown that serum placental exosomes in PE may lead to endothelial dysfunction. However, current studies mainly focus on exosomes derived from the placenta that are in the maternal circulation, and exosomes derived from the placenta in umbilical cord blood have yet to be studied.

The “Barker hypothesis” postulates that many organ structures and functions undergo programing during embryonic and fetal development. It is plausible that poor intrauterine environments, including exposure to developmental toxicants, may similarly alter adult disease susceptibility (16). Previous studies have shown that the offspring of women with gestational hypertension are at risk for clinical cardiometabolic events later in life, including hypertension and stroke (5). Study has shown that placental dysfunction is associated with cardiovascular disease (17). One study has shown that umbilical cord placental exosomes in women with PE disrupt the normal function of vascular endothelial cells by targeting HMGCS1, which may lead to vascular disease in offspring (18).

Thus, we hypothesize that H/R-exo and PE-exo damage HUVECs, resulting in endothelial cell dysfunction *in vitro*. In our study, we confirmed that H/R-exo and PE-exo led to endothelial cell dysfunction. These findings may shed light on vascular diseases of fetal origin and the pathogenesis of PE.

## 2 Materials and methods

### 2.1 Sample collection

Samples were obtained from healthy pregnant women and PE patients from the Department of Obstetrics and Gynecology of Shandong Provincial Hospital Affiliated to Shandong University from January 2020 to December 2021. The normal control pregnancy group was defined based on the absence of pregnancy complications (*n* = 10, 34.2 weeks). The PE group was defined as based on maternal blood pressure (≥140/90 mmHg) and urinary protein (≥2+) after 20 weeks of gestation (*n* = 10, 32.6 weeks), and women with diabetes and other pregnancy complications were excluded (19).

After collecting whole cord blood in the anticoagulant ethylenediaminetetraacetic acid–K2 (EDTA-K2), and centrifuging the sample at 3,000 × *g* for 20 min at 4°C, umbilical cord blood plasma was obtained and stored at −80°C for further analyses. The protocols were approved by the Ethics Committee of Shandong Provincial Hospital Affiliated to Shandong University, and informed consent was obtained from all participants.

The clinical characteristics of the patients in this study are recorded in Table 1.

**TABLE 1:** Clinical information of the study population.

Demographic	Normal (*n* = 10)	Preeclampsia (*n* = 10)
Maternal age, y	34.2 ± 3	32.60 ± 4
Gestational age at delivery, w	38.64 ± 0.1213	31.16 ± 0.7134^a^
Birth weight, g	3,355 ± 349.2	1,436 ± 464.7^a^
Systolic blood pressure, mmHg	109.7 ± 9.933	176.5 ± 10.97^a^
Diastolic blood pressure, mmHg	71 ± 3.141	110.9 ± 3.469^a^
Proteinuria	–	≥2+^a^

The data are expressed as the mean ± SD. PE vs. normal pregnancy.

^a^*P* < 0.001.

### 2.2 Cell culture

Human umbilical vein endothelial cells were isolated from three donors. The samples were treated within 2 h after collection. All samples were collected from 28 year-old women with full-term, singleton pregnancies without complications. HUVECs were digested by trypsin and cultured in endothelial cell medium (ScienCell, San Diego, CA) containing 5% fetal bovine serum, 1% penicillin/streptomycin and 1% endothelial cell growth additive at 37°C and 5% CO_2_ (20).

The HTR-8/SVneo cell line (human first trimester extravillous trophoblast cells) was purchased from ATCC; Manassas, VA. The cells were routinely cultured in RPMI 1640 (Gibco; Grand Island, State of New York, USA) supplemented with 10% fetal bovine serum (FBS). Hypoxia/reoxygenation (H/R) was performed as previously described (10). The trophoblast cells were cultured in two cycles: The first was in a hypoxic environment in a tri-gas cell culture incubator flushed with 2% O_2_ for 8 h, followed by reoxygenation in a standard incubator with 20% O_2_ for 16 h.

### 2.3 Exosome isolation and identification

#### 2.3.1 Exosome isolation from culture medium

Exosomes were isolated from cell-free HTR-8/SVneo conditioned media as previously described (21). In brief, there were two groups with different oxygenation levels: One group was subjected to 20% O_2_, 5% CO_2_, and 75% N_2_ (Non-treated, NO), and the other group was subjected to H/R as previously described (7, 8). The culture supernatants were sequentially centrifuged at 500 × *g* for 10 min at 4°C, 2,000 × *g* for 30 min at 4°C and 12,000 × *g* for 45 min at 4°C. The resultant supernatant was passed through a 0.22 μm Steritop™ filter for sterilization (Millipore, Billerica, MA, USA). Subsequently, the cleared supernatants were ultracentrifuged at 120,000 × *g* (Hitachi CP100MX, Japan) for 70 min at 4°C, and the pellets were collected. Finally, the pellet suspension was ultracentrifuged for a second time at 120,000 × *g* for 70 min at 4°C and the pellets were resuspended in 200 μl 1X PBS (13, 21). The concentration of exosomes was measured with a BCA protein analysis kit (Solarbio, Beijing, China).

#### 2.3.2 Placental exosomes isolated from umbilical cord plasma

Placental exosomes were obtained by ultracentrifugation combined with density gradient centrifugation. Briefly, after myometrium incision and placenta delivery, 20 ml of blood was collected with a sterile needle. The collected umbilical cord blood was immediately centrifuged at 4°C for 3,000 × *g* 10 min to obtain 8 ml of plasma. Next, plasma was diluted with an equal volume of PBS (pH 7.4) and then centrifuged at 500 × *g* for 10 min at 4°C. Then, the supernatant fluid was centrifuged at 2,000 × *g* for 30 min at 4°C. Subsequently, this supernatant fluid was centrifuged at 12,000 × *g* for 45 min at 4°C. The resultant supernatant was passed through a 0.22 μm Steritop™ filter (Millipore, Billerica, MA, USA). Then, the suspension was resuspended in 0.25 M sucrose, distributed on a linear sucrose density gradient, ultracentrifuged at 200,000 × *g* (Optima XPN-100) for 16 h at 4°C and divided into six fractions: f1 (supernatant), 1.03; f2, 1.06; f3, 1.09; f4, 1.11; f5, 1.14; and f6, 1.18 g/ml. Certain fractions (f2, 1.06 and f3, 1.09) were diluted in PBS (13 ml) and then centrifuged at 200,000 × *g* for 70 min at 4°C. The pellet was resuspended in 200 μl 1X PBS and stored at −80°C (13, 15, 22). The exosomes concentration was measured with a BCA protein analysis kit (Solarbio, Beijing, China).

#### 2.3.3 Transmission electron microscopy (TEM)

Ten microlitres of the exosome samples were added to the copper wire for 1 min, and the liquid was absorbed with filter paper. Then, 10 μl of phosphotungstic acid was added to the copper wire and precipitated for 1 min, and the floating liquid was absorbed with filter paper, and dried for several minutes at room temperature. Finally, the TEM imaging results were obtained by electron microscopy imaging at 100 kV (Xiuyue Biol, Jinan, China).

#### 2.3.4 Nanoparticle tracking analysis (NTA)

First, 10 μl of exosomes was diluted with 1X PBS to 30 μl. Then, the instrument’s performance (ZetaView^®^ Nanoparticle Tracking Analyzer PMX-120) was tested with standard materials, and the exosome samples were loaded after the test was passed. Gradient dilution was carried out to avoid clogging of the injection needle. Finally, the particle size and exosomes concentration were measured by the instrument after the samples were tested (Xiuyue Biol, Jinan, China).

#### 2.3.5 Western blotting

Total protein was extracted from exosomes or HUVECs. The total protein concentration was measured with a BCA kit. Next, the proteins were separated by gel electrophoresis and then transferred to polyvinylidene fluoride membranes using electrical blotting. A total of 10 μg of total protein from each sample was used for placental exosome-specific antibodies and junction protein-specific antibodies, 15 μg of total protein from each sample was used for BIM and BAX, and 40 μg of total protein from each sample was used for BCL-2. The placental exosome-specific antibodies included: CD63 (1:1,1000; Abcam, USA), TSG101 (1:1,1000; Abcam, USA); Placenta specific antibody: and PLAP (1:1,1000; Abcam, USA); The junction protein-specific antibodies included: VE-cadherin (1:1,1000; CST, USA) and Occludin (1:1,1000; Abcam, USA); The apoptosis protein-specific antibodies included: Bax (1:1,1000; CST, USA), Bim (1:1,1000; CST, USA), and Bcl-2 (1:1,1000; CST, USA); The housekeeping antibodies included: GAPDH (1:1,1000; CST, USA). The antibodies were incubated with the blots overnight at 4°C. The blots were then incubated with HRP-conjugated goat anti-rabbit secondary antibodies (Proteintech, Rosemont, IL) at room temperature for 1 h. The immunofluorescence bands were detected with a kit (Merck Millipore, Burlington, MA) and the intensity of the bands was quantified by an Amersham Imager 600 Imaging System (GE Healthcare, Chicago, IL).

#### 2.3.6 Exosome labeling and tracking

Exosome uptake by HUVECs was analyzed, and the process was performed as follows: Exosomes were labeled with the Green Fluorescent Cell Linker Kit (PKH67, green, Sigma, USA). The labeled exosomes were filtered through a 100-kDa molecular weight cut-off hollow fiber membrane. The labeled exosomes (100 μg/ml) were incubated with HUVECs for 24 h. The cells were washed twice with 1X PBS, and then immobilized with 4% formaldehyde for 30 min. The nuclei were stained with 4′, 6-di-amidino-2-phenylindole (DAPI, blue, Solarbio, China), and the cell membrane was stained with empirical formula (phalloidin, red, Sigma, USA). Finally, the uptake of exosomes by HUVECs was imaged using ImageXpress Microconfocal with MetaXpress software (overall magnification 100 ×).

### 2.4 Cell function assays

#### 2.4.1 Proliferation was assessed by EdU staining and CCK8 assay

##### 2.4.1.1 EdU staining

EdU staining was performed according to the manufacturer’s instructions (Beyotime, Shanghai, China). Briefly, HUVECs (5 × 10^3^ cells/well) were incubated with different exosomes (NO-exo, H/R-exo, N-exo, and PE-exo, 100 μg/ml) for 24 h. EdU (10 μM/well, red) was then added to the medium and incubated for 4 h. After labeling, the cells were washed three times with 1X PBS and then fixed with 4% formaldehyde. After being incubated with glycine, the cells were washed with 1X PBS containing 0.5% Triton X-100. After staining the nuclei with DAPI (blue), proliferating cells (pink) were observed by ImageXpress Microconfocal with MetaXpress software (overall magnification 200 ×).

##### 2.4.1.2 CCK8 assay

In the exosome experiment, HUVECs (5 × 10^3^ cells/well) were cultured in 96-well plates. After 24 h of coculture with different exosomes (NO-exo, H/R-exo, N-exo, and PE-exo, 100 μg/ml), 10 μl CCK8 (Bioss, Beijing, China) solution was added to each well for 2 h at 37°C, after which the cell survival rate was measured at 450 nm.

##### 2.4.2 Cell permeability assay

The flow of Evans-blue bound to albumin across the monolayer of a functional artificial liver was measured by spectrophotometry, using a modified two-compartment model that was described previously for quantitative permeability (23). In brief, HUVECs were plated (5 × 10^4^ cells/well) transwel transwell in Transwell inserts in diameters of 0.4 μm and 6.5 mm for 3 days. Confluent monolayers were incubated with different exosomes (NO-exo, H/R-exo, N-exo, and PE-exo, 100 μg/ml) for 24 h. The inserts were washed with 1X PBS (pH 7.4), and then 0.5 ml (0.67 mg/ml) of Evans-blue-BSA (4%) diluent was added to the medium. Fresh medium was added to the lower chamber, and Evans-blue-BSA was added to the upper chamber. After 10 min, the optical density at 650 nm in the lower chamber was measured.

#### 2.4.3 Apoptosis analysis by flow cytometry

The apoptosis level was determined by a BD apoptosis detection kit (BD Biosciences). In short, HUVECs (4 × 10^5^ cells/well) were inoculated in a 25 cm square dish and incubated with different exosomes (NO-exo, H/R-exo, N-exo, and PE-exo, 100 μg/ml) for 24 h before collection. Finally, the samples were stained with PI and FITC and then analyzed by flow cytometry (BD Accuri™ C6 Plus).

#### 2.4.4 Tube formation assay

To quantitatively determine the ability of HUVECs to generate blood vessels *in vitro*, substrate glue was applied to the bottom of a 96-well plate. HUVECs (1 × 10^4^ cells/well) were added to serum-free endothelial cell culture medium after Matrigel mix (BD Bioscience) coagulation, and the cells were incubated with different exosomes (NO-exo, H/R-exo, N-exo, and PE-exo, 100 μg/ml) for 6 h. The cells were stained with calcein-acetoxymethyl ester (calcein-AM), and then photographed with an inverted microscope (Thermo, USA) for analysis, ImageJ was used to quantify tube formation.

#### 2.4.5 Cell migration assay

The migration of HUVECs was measured by Transwell inserts (Corning, USA) with 8 μm polycarbonate membranes. The process was as follows: 200 μl of serum-free medium was added to the upper chamber, and 650 μl complete medium containing 5% serum was added to the lower chamber. HUVECs (1 × 10^4^ cells/well) were incubated in the upper chamber with exosomes from the different groups (NO-exo, H/R-exo, N-exo, and PE-exo, 100 μg/ml). After 24 h of cultivation, the cells that migrated to the lower chamber were stained with crystal violet. Finally, an inverted microscope (Olympus, Tokyo, Japan) was used at a magnification of 200 × to count the average number of migrated cells.

#### 2.4.7 Quantitative RT-PCR (q-RT-PCR) analysis

After 24 h of coculturing with different exosomes (NO-exo, H/R-exo, N-exo, and PE-exo), 5 μg total RNA was extracted from treated HUVECs using an RNA extraction kit. Reverse transcription was performed using a reverse transcription kit. A StepOnePlus real-time quantitative PCR system (Invitrogen, CA, USA) was used to measure mRNA in HUVECs. The VE-cadherin primers (5′-ACGACAACTGGCCTGTGTTCAC and 3′-TG-CATCCACTGCTGTCACAGAG) yielded a 101-base pair fragment. The Occludin primers (5′-ACCCCCATCTGACTAT-GTGGAA and 3′-AGGAACCGGCGTGGATTTA) yielded a 115-base pair fragment. The GAPDH primers (5′-AGATCCCTCCAAAATCAAGTGG and 3′-GGCAGAGA TGATGACCCTTTT) yielded a 130-base pair fragment. SYBR^®^ Premix Ex Taq™ (Accurate Biotechnology, Hunan, China) was used for amplification, and gene expression was calculated with the 2^–ΔΔCT^ method (with GAPDH as an internal reference).

### 2.5 Statistical analysis

The data are expressed as the means ± SDs of the three independent experiments. ImageJ software was used for data analysis, and GraphPad Prism (ver. 7; GraphPad Software Inc., La Jolla, CA) was used for statistical analysis. Student’s *t*-tests were used to analyze differences between the two groups. *P* < 0.05 was considered statistically significant (*P < 0.05, ^**^P < 0.01, ^***^P < 0.001, and ^****^P < 0.0001).

## 3 Results

### 3.1 Hypoxia model in HTR-8/SVneo cells

**FIGURE 1:**
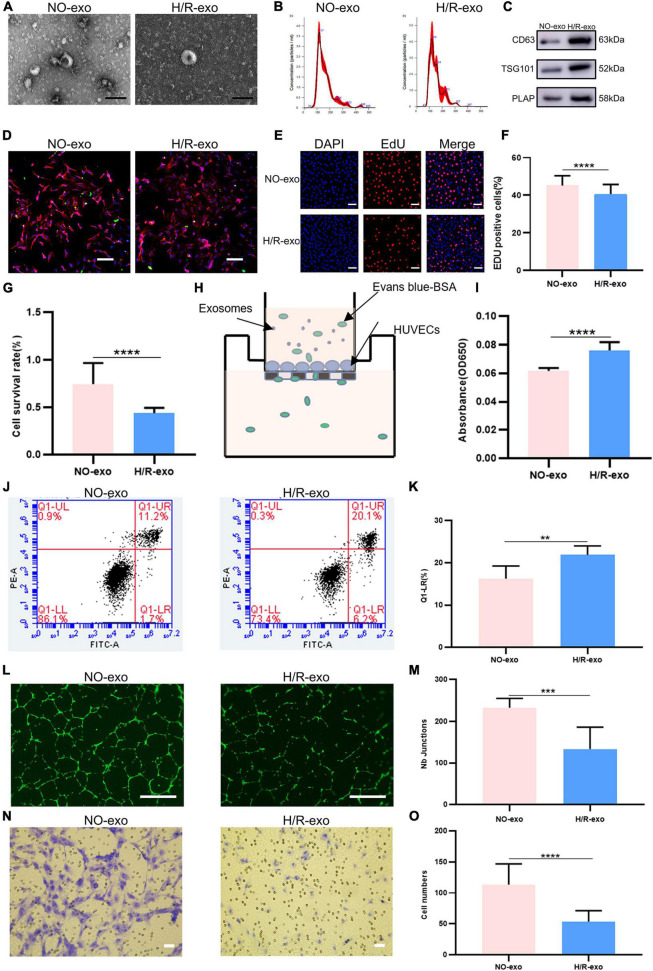
Hypoxia model in HTR-8/SVneo cells. **(A)** TEM image of exosomes from no treated (NO–exo), hypoxic/reoxygenation (H/R–exo), Scale: 100 nm; **(B)** NTA results showing the diameters of NO-exo, H/R-exo; **(C)** western blotting showed positive results for the specific exosomes markers CD63 and TSG101, and for the placental marker PLAP; **(D)** confocal microscopy showed that PKH67-labeled exosomes were internalized by HUVECs, Scale: 100 μm; **(E)** EdU staining was used to examine proliferation after exosome (100 μg/mL) treatment for 24 h, Scale: 100 μm; **(F)** EdU statistical analysis chart; **(G)** cell counting kit 8 (CCK-8) was used to examine the survival rate of endothelial cells after treatment with exosomes (100 μg/mL) for 24 h; **(H)** the endothelial monolayer barrier function of cells was tested in the model after treatment with exosomes (100 μg/ml) for 24 h; **(I)** permeability statistical analysis chart; **(J)** flow cytometry was used to examine the effect of exosomes (100 μg/mL) on the proliferation of cultured HUVECs; **(K)** apoptosis’s statistical analysis chart; **(L)** the reaction of cultured HUVECs to exosomes (100 μg/mL) was examined in the angiogenesis assay, Scale: 200 μm; **(M)** angiogenesis statistical analysis chart; **(N)** transwell experiments examined HUVECs migration after being incubated with exosomes (100 μg/mL) for 24 h, Scale: 200 μm; **(O)** migration statistical analysis chart. Each experiment was carried out three times independently. **P < 0.01, ***P < 0.001, ****P < 0.0001 comparing the same concentration of exosomes.

Non-treated-exo and H/R-exo were extracted as described previously (11, 14). The morphology and diameters of exosomes were identified by TEM and NTA, and exosomes exhibited a double-membrane cup structure with diameters of 30–150 nm (Figures 1A, B). We found no significant difference in the characteristics of NO-exo and H/R-exo. Exosomes were characterized by CD63, TSG101, and PLAP expression (Figure 1C). To determine whether exosomes could be taken up by HUVECs, exosomes were stained with PKH67, cocultured with DAPI and phalloidin-labeled HUVECs for 24 h and observed with confocal microscopy. Labeled exosomes (green) could be seen in the cytoplasms of the cells, indicating that the recipient cells could take up exosomes (Figure 1D).

EdU staining and CCK8 assays were used to examine cell proliferation. The results showed that compared with NO-exo, H/R-exo decreased cell proliferation (Figures 1E–G). Then, to investigate the effect of exosomes on the HUVECs barrier, we monitored the flux of albumin in a two-chamber system (Figure 1H). After 24 h of treatment with exosomes, the barrier was destroyed by H/R-exo, and permeability was increased compared with the effect of NO-exo (Figure 1I). In addition, cell apoptosis in response to exosomes was analyzed by flow cytometry (Figure 1J). The results showed that apoptosis in the H/R-exo group was increased compared with that in the NO-exo group (Figure 1K).

Angiogenesis experiments (Figure 1L) showed that H/R-exo attenuated the angiogenic ability of HUVECs compared with NO-exo (Figure 1M). Compared with NO-exo, H/R-exo significantly reduced the number of migrating HUVECs (Figures 1N, O). These findings suggested that H/R-exo inhibited barrier function, proliferation, angiogenesis, and migration in HUVECs, and increased cell permeability and apoptosis.

### 3.2 Preeclampsia model of oxidative stress *in vitro*

**FIGURE 2:**
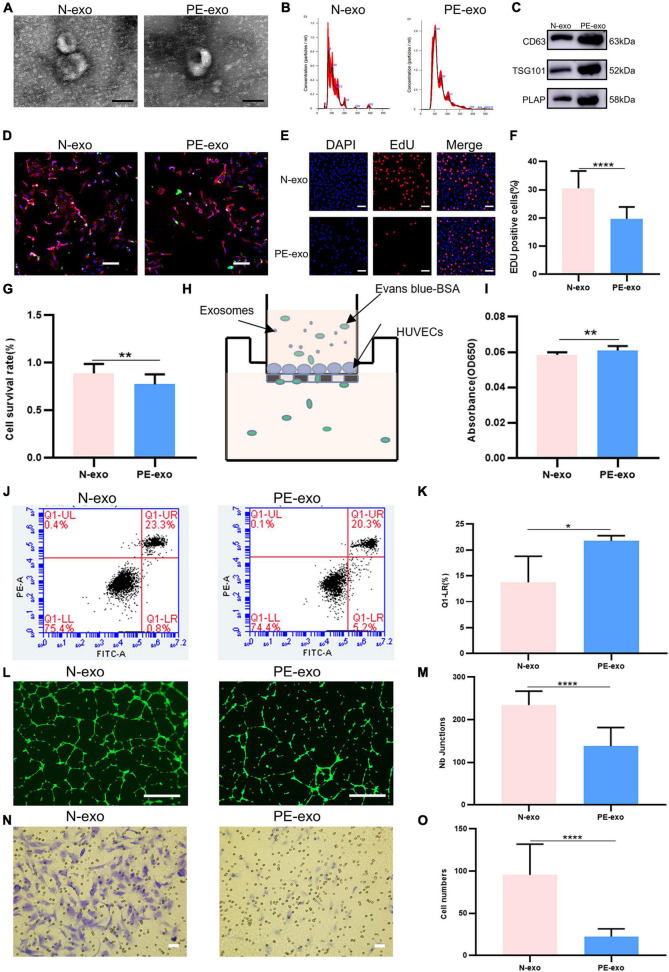
Preeclampsia model of oxidative stress *in vitro*. **(A)** TEM image of exosomes from women with normal pregnancies (N–exo) and those with early onset preeclampsia pregnancy (PE–exo). Scale: 100 nm; **(B)** NTA results showing the diameters of N-exo and PE-exo. **(C)** Western blotting showed positive results for the specific exosome markers CD63 and TSG101, and the placental marker PLAP; **(D)** confocal microscopy showed that PKH67-labeled exosomes were internalized by HUVECs, Scale: 100 μm; **(E)** EdU staining was used to examine proliferation after exosomes (100 μg/mL) treatment for 24 h, Scale: 100 μm; **(F)** EdU statistical analysis chart; **(G)** cell counting kit 8 (CCK-8) was used to examine the survival rate of endothelial cells after treatment with exosomes (100 μg/mL) for 24 h; **(H)** the endothelial monolayer barrier function of cells was tested in the model after treatment with exosomes (100 μg/ml) for 24 h; **(I)** permeability statistical analysis chart; **(J)** flow cytometry was used to examine the effect of exosomes (100 μg/mL) on the proliferation of cultured HUVECs; **(K)** apoptosis statistical analysis chart; **(L)** the reaction of cultured HUVECs to exosomes (100 μg/mL) was examined in the angiogenesis assay, Scale: 200 μm; **(M)** angiogenesis statistical analysis chart; **(N)** transwell experiments were performed to examine HUVECs migration after cubation with exosomes (100 μg/mL) for 24 h, Scale: 200 μm; **(O)** migration statistical analysis chart. Each experiment was carried out three times independently. *P < 0.05, **P < 0.01, ****P < 0.0001, comparing the same concentration of exosomes.

Table 1 shows, that compared with women with normal pregnancies (*n* = 10), women with PE (*n* = 10) had higher blood pressure and proteinuria rates, lower fetal and placental weights, and earlier weeks of termination. N-exo and PE-exo were isolated from umbilical cord blood by validated methods (15, 17, 24). TEM and NTA results showed that both exosomes had a double-membrane cup structure with diameters of 30–150 nm, and there was no obvious difference between N-exo and PE-exo (Figures 2A, B). Western blot analysis confirmed the expression of placental exosomes (CD63, TSG101, PLAP) (Figure 2C). Placental exosomes were internalized by HUVECs *in vitro*, as shown by confocal microscopy (Figure 2D).

Then, a series of cell function experiments showed that compared with N-exo, PE-exo significantly inhibited cell proliferation (Figures 2E–G), improved permeability (Figures 2H, I) and apoptosis (Figures 2J, K), and decreased tube formation (Figures 2L, M) and migration (Figures 2N, O).

### 3.3 H/R-exo and PE-exo altered the level of protein expression

**FIGURE 3:**
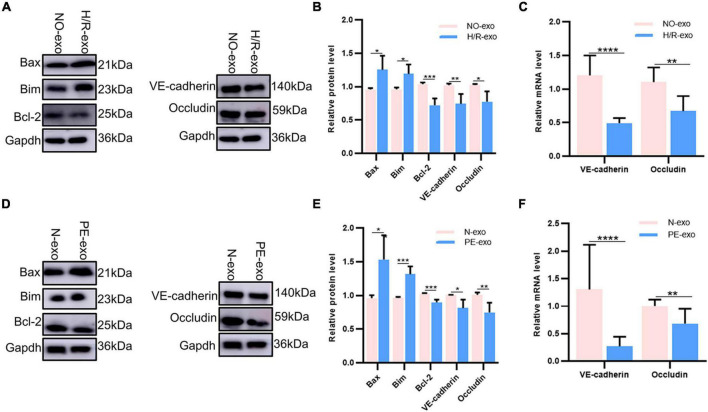
H/R-exo and PE-exo altered the level of protein expression. **(A)** Compared with NO-exo cultures, western blot showed that H/R-exo cultures significantly increased the expression of the apoptotic proteins Bax and Bim, while the expression of apoptotic protein BCI-2 and binding proteins VE-cadherin and Occludin were significantly decreased; **(B)** western blot statistical analysis chart; **(C)** compared with NO-exo, qPCR results showed that H/R-exo downregulated the mRNA expression of the binding proteins VE-cadherin and Occludin; **(D)** western blot showed that PE-exo upregulated the expression of the apoptotic proteins Bim and Bax, and downregulated the expression of the apoptotic protein Bcl-2 and binding proteins VE-cadherin and Occludin, compared with N-exo; **(E)** western blot statistical analysis chart; **(F)** compared with N-exo, qPCR results showed that PE-exo downregulated the mRNA expression of the binding proteins VE-cadherin and Occludin. Each experiment was carried out three times independently. *P < 0.05, **P < 0.01, ***P < 0.001, ****P < 0.0001.

Our experiments showed that H/R-exo and PE-exo damaged the barrier functions, proliferation, angiogenesis and migration of HUVECs, and promoted apoptosis. Therefore, we investigated the changes in apoptotic proteins levels, binding protein levels and mRNA levels. Western blot analysis showed that compared with culture with NO-exo, cultures with H/R-exo, significantly increased the expression of the apoptotic proteins Bax and Bim, while the expression of the apoptotic protein BCI-2, and the binding proteins VE-cadherin and Occludin was significantly decreased (Figures 3A, B). Next, qPCR results showed that H/R-exo downregulated the mRNA levels of the binding proteins VE-cadherin and Occludin in HUVECs compared with N-exo (Figure 3C). Western blot analysis showed that PE-exo upregulated the expression of the apoptotic proteins Bax and Bim and downregulated the expression of the apoptotic protein BCI-2 and binding proteins VE-cadherin, Occludin compared with N-exo (Figures 3D, E). Then, qPCR results showed that PE-exo downregulated the mRNA levels of the binding proteins VE-cadherin and Occludin in HUVECs compared with N-exo (Figure 3F).

## 4 Discussion

Preeclampsia can lead to fetal dysplasia, which is the main cause of adult disease (25, 26). Nevertheless, the mechanism is unclear. Placental exosomes play a key role in “mother-placenta-foetal communication.”

Preeclampsia, a common and serious pregnancy complication, has a global incidence of 3–5% and results in increased fetal and maternal morbidity and mortality (24). Studies have shown that the biological activity of exosomes from the placenta is different between normal and complex pregnancies, and the contents of exosomes are altered (27, 28). However, the function of umbilical cord blood placental exosomes in pregnancy-related diseases is still not fully understood.

Previous studies have suggested that, some diseases originate in the embryonic period (29). Studies have shown that PE may be a risk factor for offspring disease (30, 31). For example, the children of pregnant women with PE have an increased risk of neurological and cardiovascular disease compared to those of healthy pregnant women (1). HUVECs have been widely used to investigate cellular models in which PE regulates abnormal fetal endothelial function (32, 33).

**FIGURE 4:**
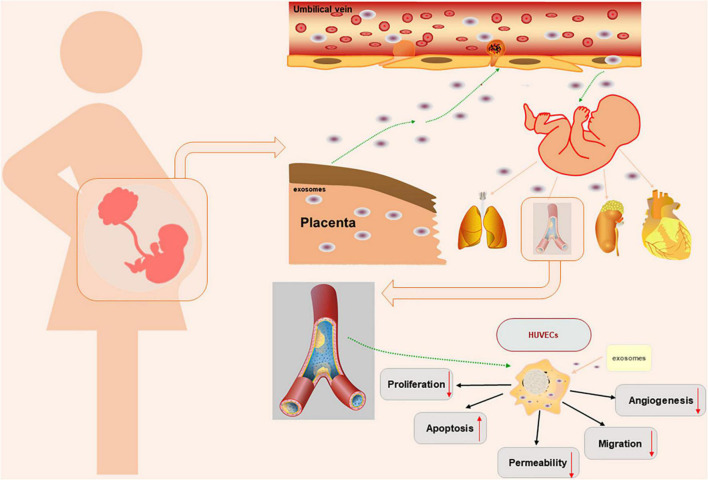
Schematic diagram of the experimental process.

Endothelial barrier function is critical for maintaining the normal physiological function of endothelial cells. Endothelial permeability is a marker of endothelial dysfunction. Studies have shown that oxidative stress in trophoblasts is involved in the pathophysiological process of PE (8, 34). The medium of hypoxic trophoblasts can increase the permeability of the endothelial monolayer. Impaired barrier function can lead to increased endothelial permeability, resulting in protein leakage, oedema, inflammation, and other responses (35). Previous experiments have shown differences in the distribution of connexins, the expression of VE-cadherin and Occludin and the permeability of endothelial monolayer cells isolated from women with normal pregnancy and preeclampsia patients. In endothelial cells isolated from patients with PE, VE-cadherin, and Occludin were disordered in cell junctions, and intercellular space was present in the cell contact area. In addition, the expression of VE-cadherin and Occludin decreased, and the permeability of the monolayer endothelium increased, which may be related to changes in the distribution of endothelial junction proteins. In addition, experiments have confirmed that increased endothelial cell permeability can promote the metastasis of tumor cells (4, 36, 37). Studies have confirmed that exosome concentrations in PE patients are slightly elevated, and trophoblasts secrete more exosomes under oxidative stress than in women with normal pregnancy (2, 38, 39). However, we did not observe significant differences in the size or concentration of exosomes under physiological and pathological conditions. Studies have shown that exosomes can participate in maternal-fetal communication and impair HUVEC functions (2, 20). Previous studies have focused on the effects of placental exosomes effects on mothers by extracting maternal blood. In this study, we successfully extracted placental exosomes from umbilical cord blood by a combination of sucrose density gradient centrifugation and overspeed centrifugation. Then, we demonstrated that placental exosomes can cause HUVEC dysfunction through a series of *in vitro* experiments (Figure 4). In this study, we first introduced the concept of “mother-placenta-foetal communication” into the study of the mechanism of fetal dysplasia caused by PE, and provided new practical support for the DOHaD (Developmental Origins of Health and Disease) doctrine.

However, this study has some shortcomings. We only established a cell model of PE under H/R conditions, and did not refine an animal model of PE. Our study was only conducted at the cellular level without tissue or animal experiments, so it was impossible to determine whether H/R-exo and PE-exo caused adverse pregnancy outcomes. It has been established that exosomes produced in normal and pathological pregnancies carry different contents (39–42). However, the present study did not further investigate the downstream effects of H/R-exo and PE-exo mediated vascular dysfunction, and did not reveal what is involved in regulating endothelial function. Therefore, how PE-exo affect endothelial cell function and the mechanisms involved need to be further elucidated. In future studies, we will sequence placental exosomes to explore what substances causes these changes and the mechanisms involved. We will conduct animal experiments to explore the effects on maternal and fetal outcomes.

## Data availability statement

The original contributions presented in this study are included in the article/supplementary material, further inquiries can be directed to the corresponding authors.

## Ethics statement

The Ethical Committee of Shandong Provincial Hospital Affiliated to Shandong First Medical University approved this study and all patients who agreed to participate in the study signed written informed consent.

## Author contributions

LL designed the study. MG performed the research. MG and QZ analyzed the data and wrote the manuscript. YW revised the manuscript. FZ, PC, XJ, SW, YL, and QL collected the clinical blood and umbilical cord samples. All authors contributed to the article and approved the submitted version.
